# Children and Parent’s Attitude and Preferences of Dentist’s Attire in Pediatric Dental Practice

**DOI:** 10.5005/jp-journals-10005-1293

**Published:** 2015-08-11

**Authors:** Vijaya Prasad Kamavaram Ellore, Mudasser Mohammed, Mahanthesh Taranath, Naveen Kumar Ramagoni, Vinod Kumar, Gururaj Gunjalli

**Affiliations:** Professor and Head, Department of Pediatric and Preventive Dentistry Navodaya Dental College and Hospital, Raichur, Andhra Pradesh, India; Former Postgraduate Student, Department of Pediatric and Preventive Dentistry Navodaya Dental College and Hospital, Raichur, Andhra Pradesh, India; Proessor, Department of Pediatric and Preventive Dentistry Navodaya Dental College and Hospital, Raichur, Andhra Pradesh, India; Reader, Department of Pediatric and Preventive Dentistry Navodaya Dental College and Hospital, Raichur, Andhra Pradesh, India; Reader, Department of Pediatric and Preventive Dentistry Navodaya Dental College and Hospital, Raichur, Andhra Pradesh, India; Senior Lecturer, Department of Pediatric and Preventive Dentistry Navodaya Dental College and Hospital, Raichur, Andhra Pradesh, India

**Keywords:** Dentist attire, Pediatric dentistry, White coat.

## Abstract

**Background:** Before arrival into doctor’s clinic, child might have acquired an impression of a clinical environment and doctor’s appearance. Different kind of doctor’s attire may evoke different reactions. By understanding children and parent’s perception and preferences about dentist’s attire, a suitable dress code could be adopted to establish good rapport with children.

**Aim:** To evaluate children and parental perceptions and preferences towards dentist attire.

**Materials and methods:** A questionnaire designed with series of photographs of male and female dental students in different attires was responded by 150 parents aged 29 to 63 years and 150 children aged 9 to 13 years.

**Results:** Seventy percent of children participants (n = 104) and 42% of parents participants (n = 63) favored the traditional white coat attire. However, 58% parents (n = 87) significantly preferred non-white coat attires in comparison to 30% of children (n = 46) (χ^2^ = 21.61, p < 0.001). No statistical significant difference was noted among the children and the parents, both favoring the use of protective wear (χ^2^ = 0.99 p > 0.05), no-significant association was found between parents and children response to white coat (χ^2^ = 0.39, p = 0.53). A highly significant difference was found between the male participants, who favored the male dentist and female participants preferring the female dentist (χ^2^ = 47.16, p < 0.001).

**Conclusion:** Our study attempted to rule out the stereotyped concept of ‘white coat fear’ among children, both children and parents favored traditional white coat attire, contrary to popular misconception ‘white coat syndrome’. However, use of child friendly attires could be useful in anxious children for better practice management.

**How to cite this article:** Ellore VPK, Mohammed M, Taranath M, Ramagoni NK, Kumar V, Gunjalli G. Children and Parent’s Attitude and Preferences of Dentist’s Attire in Pediatric Dental Practice. Int J Clin Pediatr Dent 2015;8(2):102-107.

## INTRODUCTION

Child dental anxiety has been commonly associated with the dental treatment and a visit made by a child is no less than an extraordinary event. However, its etiology is not completely known, it remains a major barrier to dental care.^1^ According to the three-pathway theory of Rachman, children may develop an anxious response directly (by conditioning) or via more indirect learning (by modeling or from information).^2^ While factors influencing anxiety vary greatly, appropriate clothing of the dentist might possibly contribute to dentist’s empathy.^3^ Barrett and Booth were the first to report a negative aspect of the traditional white coat attire, and observed that children regard formally-dressed doctors as competent but not friendly.^4^

It is important for a pediatric dentist to establish a friendly relationship with the child patients in order to subdue their pre-existing fears for delivering an effective and efficient treatment. Miller emphasized the need to reduce a patient’s anxiety and fear of dentistry as much as possible.^5^ This demands reasoning Children’s attitudes and preferences toward their dentists to obtain effect positive changes or adjustments that would make children more comfortable in a dental setup and improve the quality of dental visits. In pediatrics, concepts of ‘clown doctors’ have been observed to be effective in managing Children’s anxiety preoperatively.^6^ Cohen observed no significant difference between dentist in white jacket with shirt and tie, shirt and tie only and clinical gown and concluded that dental attire ‘has more effect on the dentist than on the patient’.^7^

More recently Mistry and Tahmassebi found parents significantly favoring the traditional white coat whereas children significantly preferred the casual attire.^8^ Kuscu et al found more children to prefer white coat attire but highlighted the use of a child friendly attire in anxious children.^9^ Accordingly, the present study was undertaken to understand the child and parent’s perception and preferences of dentist attire in order to enhance the empathy of the dentist by adopting child favorable attire.

## MATERIALS AND METHODS

A cross-sectional sample was used of all pediatric dental patients and their parents attending department of pediatric and preventive dentistry for treatment during the month of August and September. The study was approved by the Institute Ethical Committee. A total of 150 parents with a mean age of 42.61 ranging 29 to 63 years (SD ± 6.06) and 150 children with a mean age of 12.30 ranging 9 to 13 years (SD ± 0.96) participated in the study. Siblings of the children participants were excluded from the study to avoid attrition of the parents group. An informed consent was obtained from the parent of the child patient prior to distribution of questionnaire. Participants were instructed to complete the questionnaire in the waiting area prior to the start of the treatment. Single demonstrator was used to instruct the details of the questionnaire throughout the study.

*Questionnaire:* Data were collected using a specially structured pro-forma designed to gather data on gender of participants, date of birth and five statements which listed Children’s preferences of how they would prefer their dentist to be dressed accompanied with photographs of dentists wearing alternative attires.

**Figs 1A to G:**
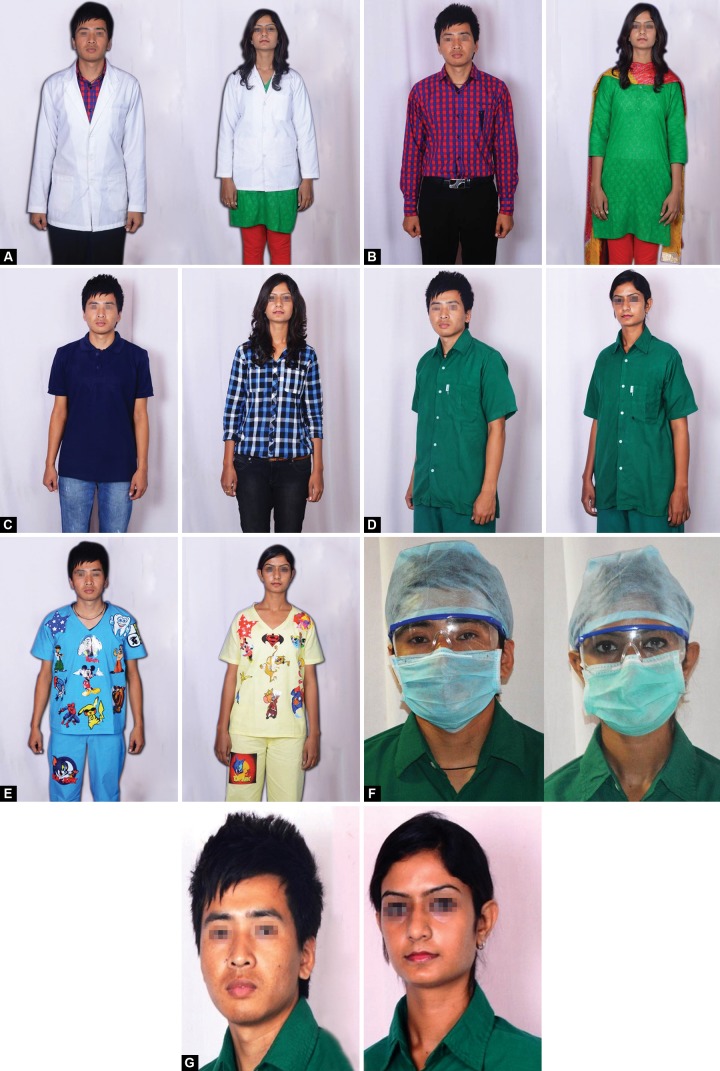
(A) Traditional white coat, (B) Formal attire, (C) Casual attire, (D) Professional attire, (E) Child friendly attire, (F) Dental student with protective gear, (G) Dental student without protective gear

*Clinical attire depiction:* Photographs of a male and female dental student in different modes of attire were taken in the photo studio using high resolution camera. Codes were allocated to each picture for ease of reference (Figs 1A to G). The attires were as follows:

- *Traditional attire:* Traditional white coat with formal shirt and formal trouser for male and formal kurti suit for female (Fig. 1A).
- *Formal attire:* Formal shirt with formal trouser for male and formal kurti suit for female (Fig. 1B).
- *Casual attire:* Casual T-shirt with casual denim jeans for male and casual shirt with casual denim jeans for females (Fig. 1C).
- *Professional attire:* Green clinical scrubs for male and female (Fig. 1D).
- *Child friendly attire:* Colored uniform with cartoon images for male and female (Fig. 1E).
- Male and female dentist with head cap, face mask and visor (Fig. 1F).
- Male and female without protective gear (Fig. 1G).

The students volunteered for the photographs treated none of the participants in the study.

## STATISTICAL ANALYSIS

All data were recorded using a SPSS® 16.0 program. Pearson Chi-squared analysis was used to determine the relationship between different variables within the study. A p-value less than or equal to 0.05 (p < 0.05) was considered as statistically significant.

## RESULTS

**Graph 1:**
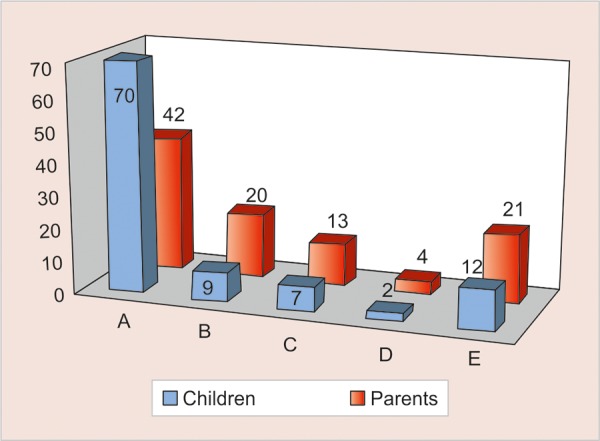
Distribution of children and parents based on attire preferences

Of the total children (n = 150) and parents (n = 150), the most popular mode of attire was the traditional white coat attire 70% (n = 104) and 42% (n = 63) respectively (Graph 1). However, we found only 34% (n = 50) of the Children’s dentists wear the white coat on a regular basis.

**Graph 2:**
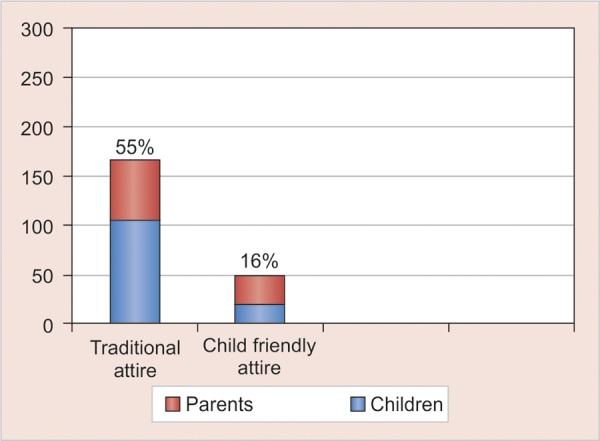
Distribution of total participants in traditional and child friendly attire groups

**Graph 3:**
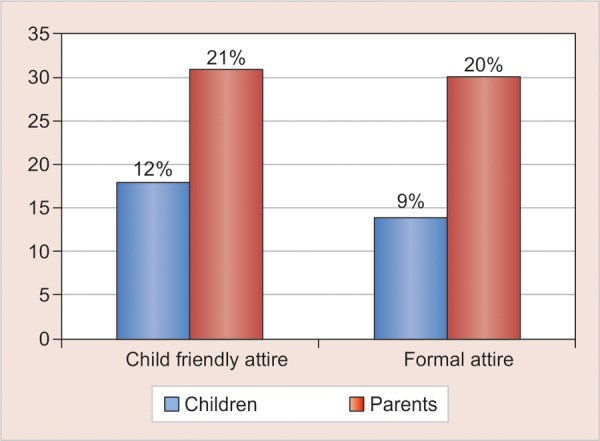
Distribution of children and parents in child friendly and formal attire groups

The least favored among the children and parent participants was the professional attire (2%, n = 3) and (4%, n = 6) respectively. Overall 16% (n = 49) of the total child and parent participants, preferred the child friendly attire in comparison to 55% (n = 167) for the traditional white coat attire (Graph 2). However, it is interesting to note that the child friendly attire was favored almost two times greater by the parents (21%, n = 31) then the children (12%, n = 18) (Graph 3).

**Graph 4:**
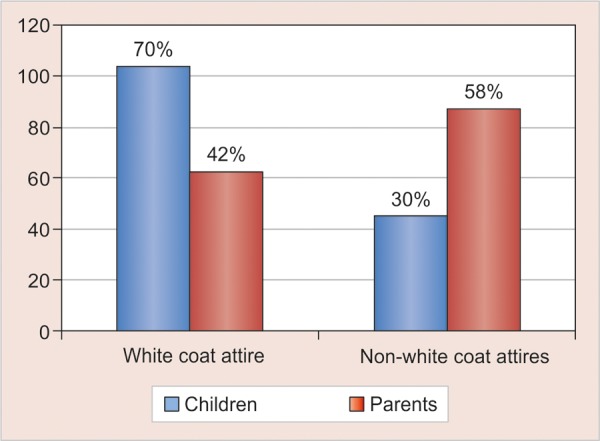
Distribution of children and parents in traditional white coat and non-white coat attire groups

**Graph 5:**
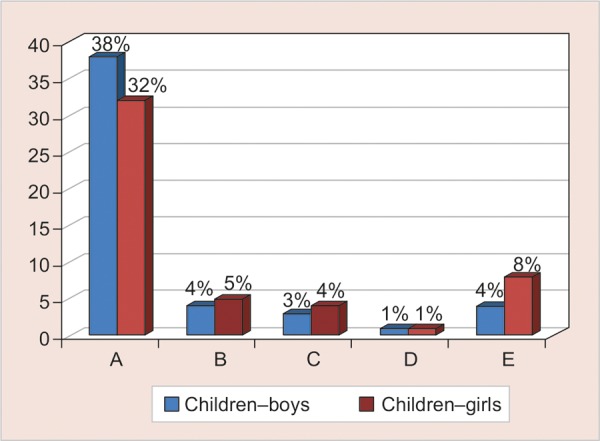
Distribution of male and female children based on their attire preferences

The same trend was noted when comparing the parents (20%, n = 30) and children (9%, n = 14) in preference toward formal attire (Graph 3). Parents (58% n = 87) showed stronger preference toward non-white coat attires in comparison with children (30% n = 46) and the difference was found to be highly significant (χ^2^ = 21.61, p < 0.001) (Graph 4). No significant difference was found between boys and girls preferences over different forms of dentist attire (χ^2^ = 3.67, p = 0.45) (Graph 5).

**Graph 6:**
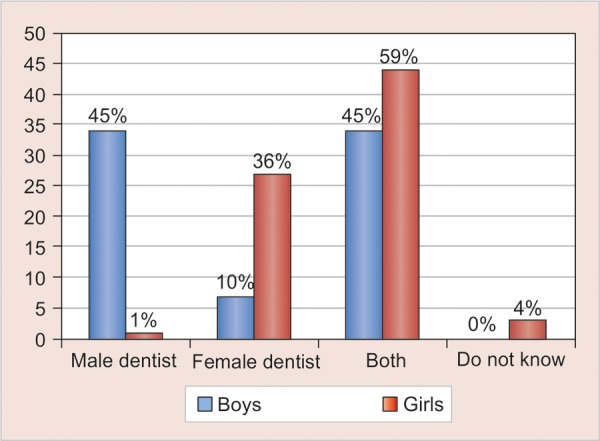
Distribution of children based on the preference of dentist’s gender

**Graph 7:**
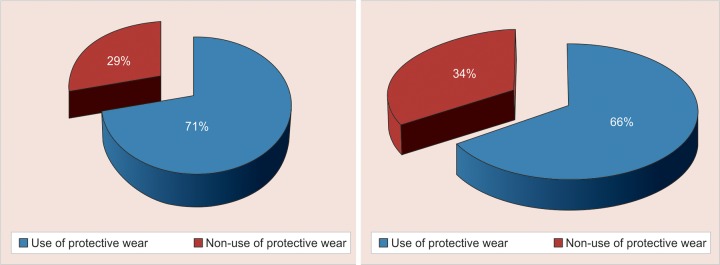
Distribution of children and parents based on their preferences of protective gear

A highly significant difference was found between the male participants (45%, n = 35) who favored the male dental student and female participants (36%, n = 27) favoring the female dental student (χ^2^ = 47.16, p < 0.001) (Graph 6). No statistical significant association was noted among the children and the parents, both favoring the use of protective wear (χ^2^ = 0.99, p = 0.32) (Graph 7).

Statistically no significant difference was found between parents and children reaction to white coat (χ^2^ = 0.39, p = 0.53) indicating a positive association between parents and children reaction on exposure to white coat.

## DISCUSSION

Earliest judgement of a dentist is made by the children based on his or her appearance, and often record and analyse their every word, movement and gesture during a dental appointment.^10^ In order to make positive changes and adjustments that would make a pediatric dentist look more acceptable for the child, importance must be given to what form of attire will be more preferential. Psychologists/sociologists highlight the importance of appearance and its effect upon first impressions and interpersonal relationships.^11^

Dunn JJ et al found physical appearance to be a vital factor in an individual’s choice of a family physician and plays an important role in the development of the physician-patient relationship.^12^ Our study revealed that both children and parents have strong perception regarding the dentist attire. The majority (70%) of the children in this study preferred their dentist’s wearing traditional white coat attire which is consistent with the results of other studies that have examined Children’s preference toward dentist attire.^8,9,13^ However, this finding differs from other studies where patients had least preference toward the white coat.^14,15^

Pediatric dentists wearing white coat must be concerned by the fact that 28% of children in this study reported that they disliked their dentist white coat and another 30% preferred the non-white coat attires for their dentist to wear. Parents in this study showed similar preference (42%) in traditional attire as reported by previous studies.^16-18^ Majority (62%) of the parents opted between traditional and formal wear which further supports the study done by Nair et al who stated parents to be more comfortable with traditional styles of appearance, such as white coats, formal suits and shirts/ tie as it gives an air of professionalism encouraging trust and confidence.^19^

However in our study, we found significant difference between children and parents selection toward traditional white coat and non-white coat attires (χ^2^ = 21.61, p < 0.001) indicating a stronger preference of white coat attire by the children and non-white coat attires by parents. This finding supports the study done by McCarthy et al that found that contrary to the common belief, children are not afraid of the physician in white coat and consider a more formally dressed physician to be more competent and concerned.^20^ Child friendly attire was the second most favored among the children (12%) and parents (21%) supporting the findings of Kuscu et al who recommended the concept of ‘Child friendly attire’ in anxious children to enhance easy first communication.^9^ Many dentists advocate the use of casual (informal) attire to make a patient more ‘comfortable’ in their surroundings the most quoted reason being to ‘avoid frightening people particularly children.^7,21^

However, our results showed that both parents (13%) and children (7%) were less likely in favor of casual attire which agrees with the findings of Kelly GR et al.^22^ Despite recent changes in western culture toward casual dress it appears that both older and younger generation continue to expect a dentist to be formally dressed. A highly significant difference in gender preference was found with males favoring male students and females female students (χ^2^= 47.16, p < 0.001) confirming the findings of Mistry et al and AlSarheed.^8,13^ Assigning children to dentists of the same gender may improve the general comfort level of children in the dentist’s office. In our study majority of the Parents (71%) and Children (66%) preferred the male and female dentist with the protective gear ( Head cap, face mask and visor) showing their awareness of potential transmucosal transmission of infective diseases and so may perceive a discernable benefit from their dental professional wearing this safety product.

Our results were in strong agreement with Shulman and Brehm who also showed that 70% child patients preferred the dentists with protective gear.^23^ McKenna et al reported that a large proportion of their participants (28%) showed no strong feelings on the use of safety glasses.^24^ Children may be intimidated by protective devices and may be unaware of the purposes they serve. Dentists may calm the fears of patients, especially children, by explaining the purpose of the protective devices.

This study has attempted to present a new insight on the misconception of ‘white coat syndrome’ which has made many dentists across the globe to hang their white coats in an attempt to enhance their relationship better with children. This was reflected in our study where we found only 34% (n = 50) of the Children’s dentist wear white coat.

In conclusion our study attempted to focus on children and parents preferences of dentist attire. We found white coat attire is most preferred by children and parents. We have introduced child friendly attire in our study and found to be second most favored among the children and parents and hence can be recommended for dentist. It is relatively easy to change one’s style of dress to suit the preferences of patients, and these changes could greatly improve a patient’s perception of the care they receive. Future research demands a larger sample size with additional age groups and different socioeconomic backgrounds that can establish a better understanding of the children preferences.
